# Bridging the immunology gap in sub-Saharan Africa

**DOI:** 10.3389/fmed.2026.1836872

**Published:** 2026-06-26

**Authors:** Mohlopheni Jackson Marakalala, Sabelo Hadebe

**Affiliations:** 1 SAMRC Center for TB Research, Division of Molecular Biology and Human Genetics, Stellenbosch University, Tygerberg, South Africa; 2 Division of Immunology, Department of Pathology, Faculty of Health Sciences, University of Cape Town, Cape Town, South Africa

**Keywords:** education, global South, immunology, low- and lower-middle-income countries, sub-Saharan Africa

## Introduction

Immunology has long served as the intellectual backbone for understanding host responses to infectious diseases. From early descriptions of antibody-mediated protection to recent systems-level analyses of immune networks, the discipline has shaped our understanding of prevention, diagnosis, and treatment of some of the world's most dreadful diseases. The development of vaccines against diseases including measles, polio, and the century-long use of the BCG vaccine to prevent severe childhood forms of tuberculosis (TB), such as TB meningitis, demonstrate proof and value in translation of immunological insights into clinical impact.

## Current teaching gaps in the global South

Yet paradoxically, in regions bearing the heaviest burden of infectious diseases—particularly sub-Saharan Africa and much of the global South—immunology often remains underrepresented in undergraduate curriculum (1, 2). This is especially striking in settings grappling with intersecting epidemics of HIV and TB, where issues such as immune deficiency, immune activation, overwhelming inflammation and immune-mediated pathology are central to clinical outcomes (1). Studies from the global North have shown that underrepresented groups tend to pick topics relevant to themselves, their values and communities, thus using their own cultural capital and utility values to find relevance of immunology in their own success. Some examples include under-represented minority students choosing research topics such as diabetes, infectious agents, systemic lupus erythematosus or multiple sclerosis as these are prevalent in their own communities and this can guide self-directed learning and critical research questions based on experience (2, 3). The limited formal teaching of immunology at undergraduate level and lack of advanced infrastructure constrain the pipeline of future scientists and clinicians equipped to interrogate and address these challenges. There is evidence from the global North on how underrepresented groups and woman can overcome obstacles and bridge the gap in immunology teaching. These examples can be crucial in the global South to retain talent and broaden access (3). Immunology is also unique in biomedical science, in that, it has its own language and terms that can feel alienating to a second or third language speaker, which is generally the case in most low and middle income countries.

## The promise in development of biomarkers and host directed therapies (HDTs)

Over the last few decades, in terms of research, immunology has grown and diversified into multiple interconnected branches: innate immunity, adaptive (humoral and cellular) responses, trained immunity, and, more recently, systems immunology driven by big data and high-dimensional technologies. Advances in transcriptomics, proteomics, and multiparameter cytometry now allow researchers to map immune responses with greater resolution, deep in tissues. These tools have catalysed a shift from descriptive immunology to disease-predictive and translational immunology, particularly in the field of diagnostic biomarker discovery. In TB, for example, blood-based biomarkers composed of cytokines, chemokines, and growth factors are being investigated to distinguish latent infection from active disease and to identify individuals at risk of disease progression. Blood based cytokines/chemokines have also been instrumental in understanding different stages of the TB spectrum, including sub-clinical TB (4). Such biomarkers are inherently immunological in origin. They reflect host-pathogen interactions and the balance between protective immunity and pathological inflammation. Without robust immunology training, researchers may lack the conceptual grounding needed to design relevant research, and interpret or translate discoveries into practice.

Beyond diagnostics, immunology underpins the emerging field of host-directed therapies (HDTs) (5). In diseases such as tuberculosis, tissue destruction is often driven not only by microbial burden but also by overwhelming inflammation. Therapeutic interventions that modulate immune pathways, rather than directly targeting the pathogen, aim to restore balance between microbial control and tissue preservation. This principle extends to chronic inflammatory conditions such as rheumatoid arthritis, multiple sclerosis and allergic disorders, where treatment is based on targeting inflammation. Dermatological manifestations, including immune-mediated skin lesions and hypersensitivity reactions, further illustrate the pervasive clinical relevance of immunology across multiple specializations.

## The need for a strong immunology training at undergraduate levels in the global South

Despite the above aspects underpinning the importance of immunology, in many countries in the global South, immunology is often embedded as a small component within larger courses such as pathology, Biochemistry or microbiology. In undergraduate medical training, immunology may account for only a fraction of assessed content, introduced early in training usually pre-clinical years and revisited briefly later. Graduates frequently recognise the importance of immunology only in retrospect—when encountering vaccination strategies in primary healthcare, allergy management, biomarker-driven diagnostics, or immunomodulatory therapies in clinical practice or research set up. By contrast, in the global North countries such as the United Kingdom, United States, European Union, immunology exists as a distinct academic discipline, with dedicated undergraduate majors and postgraduate specialisations that interface closely with locally and globally relevant diseases. In South Africa, subspecialisation exists in infectious diseases and in allergology, but immunology itself is rarely positioned as a standalone clinical or translational discipline. This structural gap underpins a need in fostering locally driven innovation. Universities such as University of Cape Town have introduced what is called intercalated programmes for Medical students in their third year of study. This program aims to introduce medical research at an early phase of learning before students enter clinical years. A number of students who take up this program go on to do Honours degrees across various disciplines in Medical science and often extend to higher degrees such as Masters and PhD with a special registration approved by the University Senate to allow them to register for multiple degrees concurrently. As of 2026, we still do not have an MD-PhD program in any of the 10 South African universities that have medical schools. In constrast, in India, institutes such as Sree Chitra Tirunal Institute for Medical Sciences and Technology (SCTIMST), Kasturba Medical College (KMC) and Indian Institute of Technology (IIT) have well established MD-PhD programs aimed at training physician scientists to bridge the gap between clinical practise and biomedical research (6, 7). In Brazil, Federal University of Rio de Janeiro established its MD-PhD program in 1995, followed by Universidade de São Paulo (USP) and Universidade Estadual de Campinas (UNICAMP) (6). While these MD-PhD programs exists, their contribution to biomedial research translation is insignificant when compared to countries like the US and Japan. There are often no national guidelines on how MD-PhD programs should be developed or how they integrate within academic of health systems and it becomes difficult to find positions that allow for both experimental and clinical work (6, 7). An alternative proposition is a PhD-MD program, where well trained biomedical scientists are trained in medicine. Such a proposition is deemed suitable and economically viable option for LMICs where evidence based decision making may be crucial in rural settings with little to no resources (7).

Immunology has a potential to serve as a conceptual bridge across biomedical sciences. Virology, for instance, depends on understanding antiviral immune responses; bacteriology and mycology require insight into pathogen-specific immunity; biochemistry intersects with immunology through kinase signalling and intracellular pathways; and oncology increasingly leverages immune checkpoint modulation and immunotherapy. In fact majority of disciplines within clinical medicine apply some level of immunological understanding to treat or diagnose or monitor disease pregression, remission or relapse. A strong immunology foundation therefore enhances coherence across curricula, integrating multiple disciplines into a unified framework of host biology.

## Ongoing efforts to bridge the gap

Encouragingly, there are some efforts in place to address these gaps. The South African Immunological Society convenes a bi- annual conference and organizes regular webinars that create platforms for clinical and basic science trainees and established scientists to exchange knowledge and create collaborative networks. At the University of KwaZulu-Natal, there is an undergraduate module in Immunology within the Biomedical Science Honours in Medical Microbiology. At the University of Cape Town, an Advanced Immunology course introduces postgraduate students, postdoctoral fellows, and clinicians to the discipline through research-oriented teaching method. Such course has also been incorporated in the training strategy in the SAMRC Centre for TB Research at Stellenbosch University. By emphasising critical reading of primary literature, experimental design, and analysis of emerging research trends, the course models immunology as a dynamic method of scientific rigor. The course is popular amongst clinical trainees in their Masters in Medicine specialisation across disciplines including paediatrics, autoimmunity, allergy and National Health Laboratory Services Pathology disciplines who are often unprepared for the diagnostics laboratory where interpretation and recommendation of tests requires an expert understanding of immunology. Another exciting initiative is Immunopaedia, an initiative affiliated with the International Union of Immunological Societies, which has expanded access to immunology education through online resources, “Immunology of the Month” features, and summer schools across sub-Saharan Africa and other global South countries (8). These initiatives demonstrate both demand and feasibility for broader immunology training.

## Future perspectives

What remains is structural integration. A dedicated Bachelor of Science in Infectious Disease Immunology, or a similarly structured undergraduate programme, could introduce foundational principles early, transition to applied immunology in intermediate years, and culminate in research-driven successful projects. Such a curriculum would prepare graduates to harness big data tools, design biomarker studies, develop host-directed therapies, and contribute to vaccine innovation tailored to local disease burdens. At the University of Cape Town, such a program is already taking shape, focusing on well-established medical education pedagogies (3) such as flipped classrooms, use of memorable visuals and conceptual tools such a interactive videos, games such as “Immunity–Stayin' Alive; CoImmunicate” and in class non-formative assessments using Mentimeter. In clinical medicine, a more prominent immunology program geared towards clinically applied immunology could set future doctors with adequate skills as practise medicine. A Health Professions Council of South Africa recognised Masters in Medicine focussed in Immunology as a discipline would be a game changer in a many disciplines including oncology, pathology, gastroenterology, autoimmunity to name a few and would open a new career path for clinicians that can work across disciplines or multi-disciplinary clinical care, almost like anesthesia. For countries disproportionately affected by infectious and inflammatory diseases, immunology could be a strategic educational necessity, where the use of cultural capital and utility values can shape student interest and success. These are very powerful methods of getting under-represented minority groups involved in immunology research, where student use their own experience from their communities to develop research questions, therapeutic solutions and patient care based on deep cultural knowledge which can aid in better understanding of diseases. Embedding immunology more robustly within undergraduate and clinical training systems would strengthen local capacity to generate diagnostics, therapeutics, and preventive strategies. In doing so, it would empower the global South not only to respond to existing epidemics but to shape the immunological agenda of the future.
